# Identifying the optimal time point for adaptive re-planning in prostate cancer radiotherapy to minimise rectal toxicity using normal tissue imaging biomarkers^☆^

**DOI:** 10.1016/j.phro.2025.100850

**Published:** 2025-10-08

**Authors:** Zhuolin Yang, David J. Noble, Sarah Elliot, Leila Shelley, Thomas Berger, Raj Jena, Duncan B McLaren, Neil G. Burnet, William H. Nailon

**Affiliations:** a Department of Oncology Physics, Edinburgh Cancer Centre, Western General Hospital, Crewe Road South, Edinburgh EH4 2XU, UK; b Institute for Imaging, Data and Communications, School of Engineering, University of Edinburgh, the King’s Buildings, Mayfield Road, Edinburgh EH9 3JL, UK; c Edinburgh Cancer Research, CRUK Scotland Centre, Institute of Genetics and Cancer, University of Edinburgh, Edinburgh, UK; d Department of Clinical Oncology, Edinburgh Cancer Centre, Western General Hospital, Crewe Road South, Edinburgh EH4 2XU, UK; e Centre for Medical Informatics, Usher Institute, University of Edinburgh, UK; f Radiation Oncology Department, Center Hospitalier Lyon Sud, Pierre Benite, France; g The University of Cambridge, Department of Oncology, Cambridge Biomedical Campus, Hills Road, Cambridge CB2 0QQ, UK; h Proton Centre, The Christie NHS Foundation Trust, Manchester M20 4BX, UK; i School of Science and Engineering, the University of Dundee, Dundee DD1 4HN, UK

**Keywords:** Machine learning, Radiomics, Adaptive radiotherapy, Prostate cancer

## Abstract

**Background and purpose:**

Adaptive radiotherapy (ART) in prostate cancer (PCa), although not yet standard practice, is typically triggered by inter-fractional anatomical changes that emerge progressively during treatment. This study investigates whether radiomics extracted before and during treatment can identify the optimal time point for re-planning, with the goal of reducing late rectal bleeding.

**Materials and methods:**

This study included 187 PCa patients from the single-centre, prospectively collected VoxTox dataset (UK-CRN-ID-13716), treated with image-guided radiotherapy using TomoTherapy and daily MVCT. Patients received either 74 Gy in 37 fractions (N = 110) or 60 Gy in 20 fractions (N = 77). Radiomic features were extracted from pre-treatment planning CTs and daily MVCTs. Grade ≥ 1 rectal bleeding was assessed at 2 years post-treatment using CTCAE v4.03. Two analysis strategies were employed: a separate analysis, where weekly features were evaluated independently; and a cumulative analysis, which progressively incorporated features from previous weeks. Logistic regression models with elastic net penalty were trained and evaluated using AUC.

**Results:**

In both groups, week 1 provided the highest standalone predictive performance (test AUC = 0.766 for 74 Gy; 0.734 for 60 Gy). In the cumulative analysis, week 3 was optimal for the 74 Gy group (test AUC = 0.767), balancing performance and timing. For the 60 Gy group, week 1 remained optimal but suffered from reduced generalisability (test AUC = 0.643).

**Conclusions:**

Radiomic analysis of daily imaging can support early, proactive ART in PCa, offering a personalised strategy to reduce late rectal bleeding beyond conventional anatomy-based approaches.

## Highlights

• Radiomic features before and during treatment predict late rectal bleeding.
• Radiomic features from week 1 showed strongest standalone predictive performance.
• Week 3 was optimal for re-planning in patients treated with 74 Gy in 37 fractions.
• Radiomics enable biologically informed adaptation beyond anatomy-based methods.
• Analysis includes both standard and moderately hypofractionated treatment regimens.

## Graphical abstract

**Figure:**
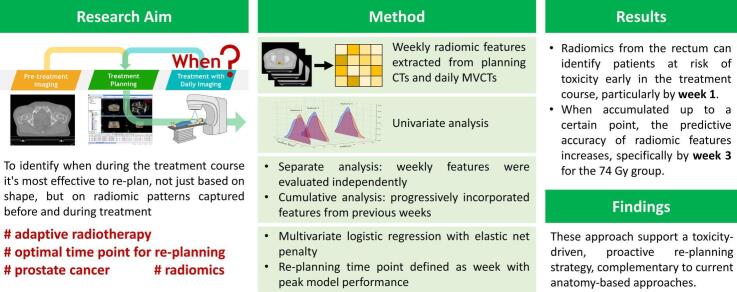

## 1 Introduction

Conventional radiotherapy for prostate cancer (PCa) is typically guided by a static treatment plan, derived from imaging acquired before the start of treatment. This plan is used throughout the treatment course without modification, and therefore does not account for inter-fractional anatomical changes such as prostate gland shrinkage, organ deformation, or shifts in the relative positioning of the prostate and adjacent organs at risk (OARs) [1]. Additional intra-fractional variations, such as bladder filling, can further displace the target and surrounding tissues. These uncertainties may lead to geographic miss or unintended irradiation of OARs, increasing the risk of toxicity [2,3].

Adaptive radiotherapy (ART) addresses these limitations by modifying treatment plans in response to patient-specific anatomical changes observed during the treatment course [4,5]. In PCa, offline ART is already used in many clinics to account for inter-fractional variability and protect OARs. Evidence supports adaptive re-planning in PCa radiotherapy when anatomical or dosimetric changes occur. Choi et al. identified thresholds of body contour variation that warrant plan adaptation [6], while Muelas-Soria et al. showed that early dose deviations predict rectal dose constraint violations [7]. Christiansen et al. demonstrated that magnetic resonance (MR)-guided ART reduces bladder and rectal doses [8]. While such approaches improve dosimetric precision, they remain reactive, typically triggered by visible and often late-occurring anatomical changes. Moreover, these studies provide little guidance on when re-planning should be performed to proactively reduce the risk of late rectal toxicity. This gap forms the basis for the present study.

Given that daily image guidance is routinely performed in PCa radiotherapy, commonly using cone-beam computed tomography (CBCT) or megavoltage CT (MVCT), there is an untapped opportunity to extract greater clinical value from these images beyond geometric verification. In particular, serial imaging may reveal early radiomic changes in rectal tissues that provide predictive insight into later toxicity risk. Previous studies have demonstrated that radiomic features from medical images can predict radiation-induced toxicity [[9], [10], [11], [12], [13], [14]], reflecting underlying tissue heterogeneity and individual radiosensitivity [15,16]. Building on this, the present study investigates whether radiomic patterns captured before and during treatment can help determine the optimal time point for adaptive re-planning, with the goal of proactively reducing late rectal toxicity.

## 2 Materials and methods

### 2.1 Patients

This study utilised data from 187 PCa patients enrolled in the VoxTox study (UK-CRN-ID-13716) [17,18], with a prospectively collected single-centre dataset recruited between March 2013 and June 2019. All patients received helical image-guided radiotherapy (IGRT) on TomoTherapy (Accuray, Sunnyvale, CA, USA) with daily guidance MVCT imaging. If excessive rectal dilation was observed on MVCTs, patients were asked to empty their rectum before treatment[17,19,20]. Acquisition parameters for planning CTs and daily MVCTs are listed in Supplementary Tables S1 and S2. Two treatment regimens were included: 110 (59 %) patients received 74 Gy in 37 fractions, and 77 (41 %) received 60 Gy in 20 fractions. Elective lymph node irradiation was delivered in ∼ 13 % patients [21]. Radiotherapy protocols have been described in previous studies [18,19,[21], [22], [23], [24]]. The endpoint of interest was grade ≥ 1 rectal bleeding at two years post-radiotherapy, assessed using the Common Terminology Criteria for Adverse Events (CTCAE v4.03) [25]. Toxicity was recorded using a standardised questionnaire developed for prostate radiotherapy research [21]. In total, 62 patients (33 %) experienced grade ≥ 1 rectal bleeding: 32 in the 74 Gy group and 30 in the 60 Gy group. Incidence rates are summarised in Table 1. This study represents a secondary retrospective analysis of the imaging and clinical data. All analyses were conducted using Python 3.10.14 (Python Software Foundation, OR, USA).

**Table 1:** Incidence rates at 2 years for 187 patients in the VoxTox prostate dataset, stratified by prescribed dose (74 Gy vs. 60 Gy).

Toxicity Endpoint	Total patients,N (%)	74 Gy,N (%)	Training set,N (%)	Test set,N (%)	60 Gy,N (%)	Training set,N (%)	Test set,N (%)
	187 (100^a^)	110 (59^a^)	82 (75^b^)	28 (25^b^)	77 (41^a^)	58 (75^e^)	19 (25^e^)
Rectal Bleeding							
= G0	125 (67^a^)	78 (71^b^)	58 (71^c^)	20 (71^d^)	47 (61^e^)	36 (62^f^)	11 (58^g^)
≥ G1	62 (33^a^)	32 (29^b^)	24 (29^c^)	8 (29^d^)	30 (39^e^)	22 (38^f^)	8 (42^g^)
= G1	41 (22^a^)	22 (20^b^)	16 (20^c^)	6 (21^d^)	19 (25^e^)	14 (24^f^)	5 (26^g^)
= G2	18 (10^a^)	9 (8^b^)	7 (9^c^)	2 (7^d^)	9 (12^e^)	6 (10^f^)	3 (16^g^)
= G3	3 (2^a^)	1 (1^b^)	1 (1^c^)	0	2 (3^e^)	2 (3^f^)	0

Note:

a. The percentage is calculated using the total number of patients (N = 187) as reference.

b. The percentage is calculated using the number of patients in the 74 Gy group (N = 110) as reference.

c. The percentage is calculated using the number of patients in the training set assigned to the 74 Gy group (N = 82) as reference.

d. The percentage is calculated using the number of patients in the test set assigned to the 74 Gy group (N = 28) as reference.

e. The percentage is calculated using the number of patients in the 60 Gy group (N = 77) as reference.

f. The percentage is calculated using the number of patients in the training set assigned to the 60 Gy group (N = 58) as reference.

g. The percentage is calculated using the number of patients in the test set assigned to the 60 Gy group (N = 19) as reference.

### 2.2 Radiomic features

Radiomic features were extracted using in-house Python software compliant with the Image Biomarker Standardization Initiative (IBSI) [26], generating a total of 118 features (Supplementary Table S3).

**Fig. 1:**
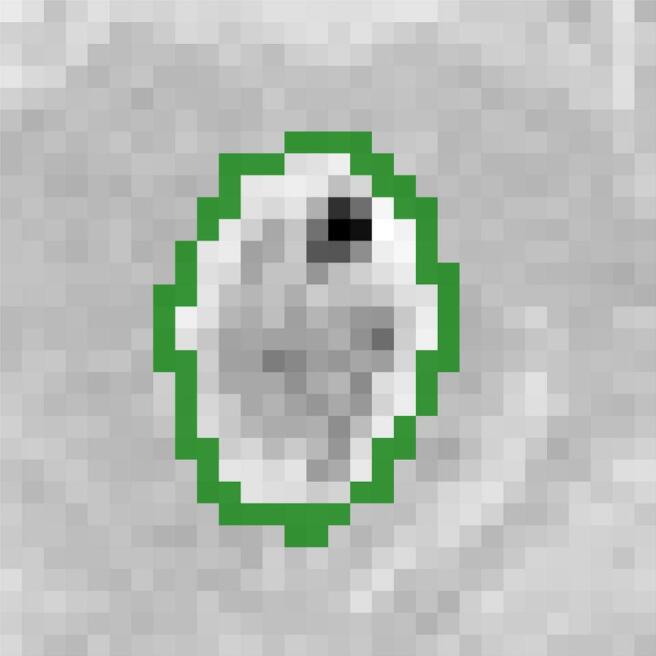

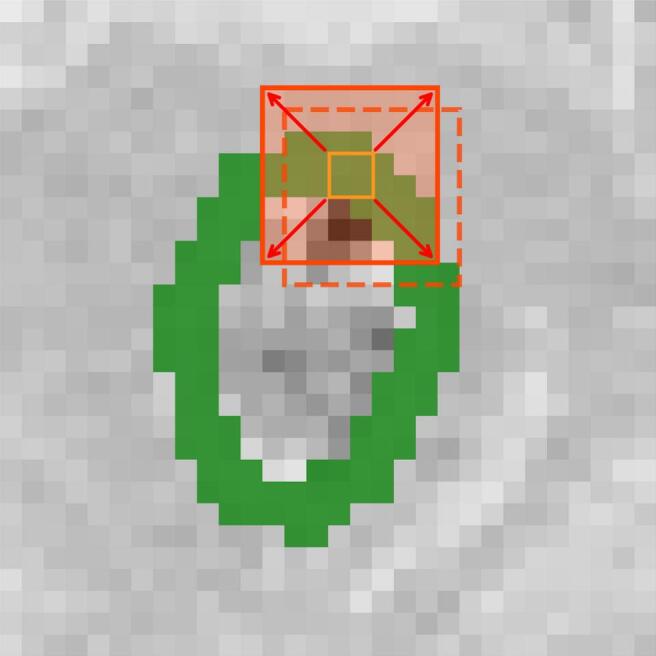
Example of rectal wall subimages extraction using planning CT. (a) The manually delineated rectum (green), (b) the resulting 2-pixel-wide rectal wall region of interest (green) after inward expansion, with surrounding 8x8 pixel^2^ subimages (orange squares) extracted at 1-pixel intervals by expanding the ROI anteriorly and posteriorly by 3 pixels. (For interpretation of the references to colour in this figure legend, the reader is referred to the web version of this article.)

The rectal wall was delineated on each slice by expanding the rectum contour inwards by 2 pixels. Subsequently, 8x8 pixel^2^ subimages were extracted at 1-pixel intervals, further expanding the 2-pixel-wide rectal wall by 3 pixels both anteriorly and posteriorly (Fig. 1) [10]. For planning CTs, features were derived from these subimages and averaged across all subimages and slices, while for daily MVCTs, features were aggregated on a weekly basis. Specifically, for the 74 Gy group (37 fractions), the MVCT radiomic features were grouped into 7 weekly segments (week 1: fractions 1–5, week 2: fractions 6–10, week 3: fractions 11–15, week 4: fractions 16–20, week 5: fractions 21–25, week 6: fractions 26–30, week 7: fractions 31–37). For the 60 Gy group (20 fractions), data were organised into 4 weeks (week 1: fractions 1–5, week 2: fractions 6–10, week 3: fractions 11–15, week 4: fractions 16–20). The MVCT radiomic features for each patient were then averaged across these defined weeks to generate the week-level features.

### 2.3 Analysis

Patients (N = 187) were stratified by prescription dose: 74 Gy (N = 110) and 60 Gy (N = 77). Each group was further split into training set and test sets with a 75:25 ratio (Table 1). All features were z-score standardised (mean = 0, std = 1), where parameters were calculated from the training set and applied to both training and test sets.

**Fig. 2:**
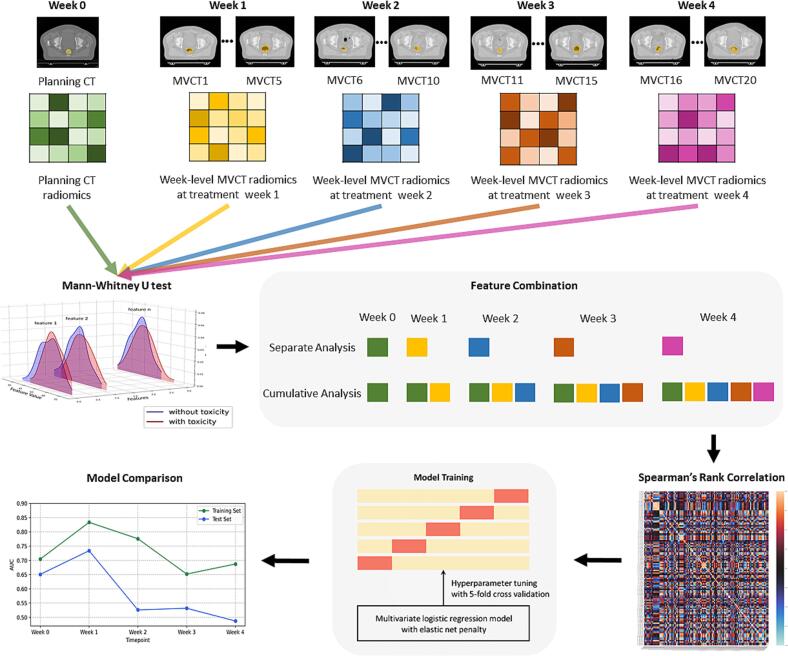
Workflow used in the analysis of radiomic features for predicting the optimal time point to adapt radiotherapy, using the 60 Gy group as an example.

The analysis process began with the Mann-Whitney *U* test to examine the relationship between individual radiomic features from the training set and rectal bleeding. This univariate analysis served primarily to highlight features that were statistically different between patients with toxicity and patients without toxicity, but did not inherently select features for the prediction model. Features from different time points were analysed both separately and cumulatively to understand their individual and combined impact on predicting the endpoint. In the separate analysis, features from distinct time points were evaluated independently to assess their standalone predictive power. Conversely, in the cumulative analysis, features from preceding time points were combined to enrich the predictive model, which allowed for a more comprehensive evaluation of how accumulated radiomic data from multiple stages influenced the outcome, providing a deeper insight into the dynamic changes occurring throughout treatment and their cumulative effects on mild rectal toxicity. To refine the selection of features and manage the dimensionality of the data, Spearman’s rank correlation coefficient was calculated for all pairs of features for each time point and for different analysis methods. For any pair with correlation ≥ 0.8, the feature with the less statistical significance in its relationship with the endpoint was removed from consideration. Each feature set trained a multivariate logistic regression model with an elastic net penalty that allowed further feature selection during training [27]. Five-fold cross-validation was used to internally tune the hyperparameters, specifically the regularisation strength (C, ranging from 10^-4^ to 10^3^ on a logarithm scale with 500 intervals), with the elastic net mixing parameter (L1 ratio) fixed at 0.5. Model performance was evaluated using area under the curve (AUC) values with 95 % confidence intervals (CIs) derived from 100 bootstrap samples. Sensitivity and specificity were assessed at the optimal threshold determined by the Youden Index [28]. The predictive performance of different models was evaluated and compared using one-sided Mann-Whitney U rank tests based on the AUC values. The analysis workflow is shown in Fig. 2.

## 3 Results

In the univariate analysis of grade ≥ 1 rectal bleeding across both the 74 Gy and 60 Gy groups, only a minimal number of radiomic features demonstrated p-values below the typically accepted threshold of 0.05. Specifically, in the 74 Gy group, features extracted from MVCTs during weeks 1, 5, and 7 did not yield any statistically significant findings. Similarly, in the 60 Gy group, weeks 1, 2, and 3 also lacked statistically significant features, reflecting the independent predictive utility of radiomic features at different time points. The top 10 features with the smallest p-values for each time point are provided in Supplementary Table S4.

In the separate analysis, the number of radiomic features selected after Spearman's rank correlation varied by time point. For the 74 Gy group, the counts were: 23 features from planning CT, 10 from week 1, 12 from week 2, 11 from weeks 3 and 4, 10 from weeks 5 and 6, and 9 from week 7. For the 60 Gy group, the counts were: 25 features from planning CT, 12 from week 1, 11 from weeks 2 and 3, and 13 from week 4. Features selected by the pairwise Spearman’s Rank Correlation for each time point in the separate analysis are given in Supplementary Table S5. In the cumulative analysis, the features selected in the separate analysis at each time point were carried forward and accumulated, which means that the features from each successive week were built upon those identified in previous weeks, maintaining a consistent and expanding basis for predicting toxicity as the treatment progresses.

**Fig. 3:**
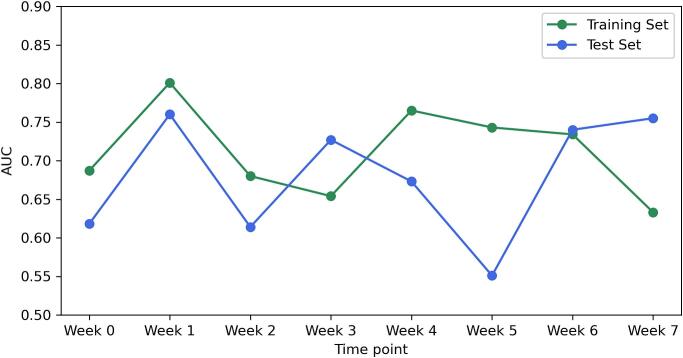

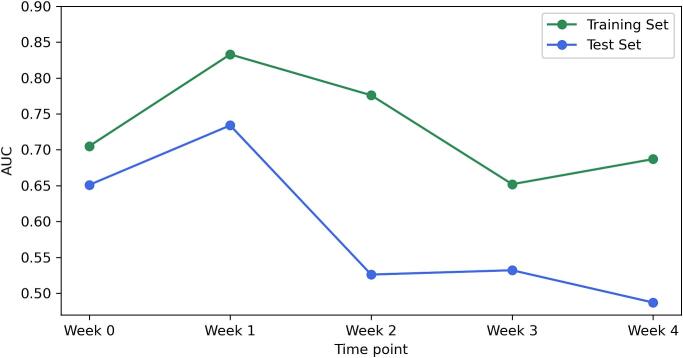

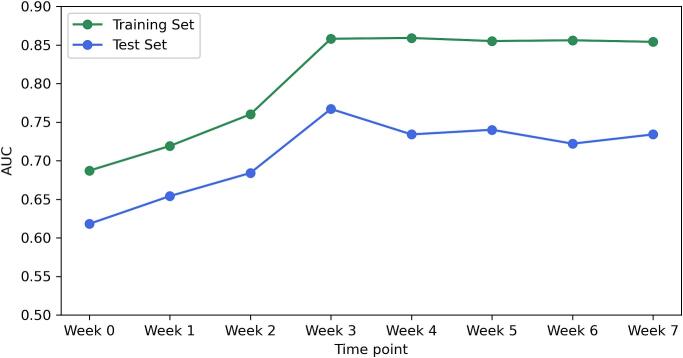

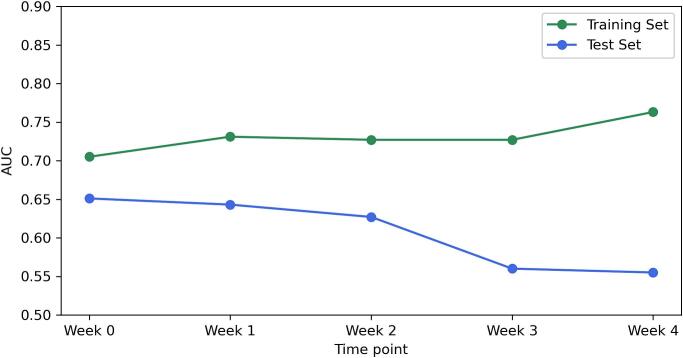
Overview of logistic regression model performance across treatment time points: these figures present the AUC performance for separate and cumulative analyses of radiomic data in predicting the optimal time point to adapt prostate cancer radiotherapy. Each subfigure illustrates the dynamic evolution of model accuracy over the course of treatment, emphasising the temporal effectiveness of radiomic features in predicting treatment outcomes. (a) Separate analysis for the 74 Gy group, (b) separate analysis for the 60 Gy group, (c) cumulative analysis for the 74 Gy group, and (d) cumulative analysis for the 60 Gy group.

Fig. 3 displays the AUC performance over the treatment course. For both groups, the separate analysis using logistic regression revealed notable differences in predictive performance across different time points. In the 74 Gy group, week 1 emerged as the most effective time point, showing the highest AUC values for both training (AUC = 0.801) and test sets (AUC = 0.766). Similarly, in the 60 Gy group, week 1 also demonstrated optimal performance with the highest AUC on both training and test sets (Training AUC = 0.833, Test AUC = 0.734). For the cumulative analysis, the AUC values for the 74 Gy group increased progressively until week 3, after which they plateaued. While week 4 exhibited the highest training AUC of 0.859, week 3 was determined to be the optimal time point for adapting treatment strategies, given its earlier occurrence and the lack of significant improvement in subsequent weeks. For the 60 Gy group, week 1 was identified as the optimal time point to adapt treatments. Despite week 4 showing the best training AUC of 0.763, its test AUC was comparatively lower (0.555), and considering it is the last week of radiotherapy, its practical utility is limited. Table 2 presents the best-performing logistic regression model for each treatment group, derived from separate and cumulative analyses. These best models were selected based on both the training AUC values and the treatment timing. This table only presents parameters that retained non-zero values after applying the elastic net penalty during training, focusing on meaningful predictors of treatment outcomes. Differences between the selected best models and models from other time points are further evaluated through p-values.

**Table 2:** Results of the multivariate logistic regression models with details.

**Model/** **Time point**	**Parameter**	**Coefficient**	**P-Value**	**Train** **AUC(95 % CI)**	**Train** **SEN(95 % CI)**	**Train** **SPE(95 % CI)**	**Highest P-Value** **(vs. Other Model/Timepoint)**	**Test** **AUC(95 % CI)**	**Test** **SEN(95 % CI)**	**Test** **SPE(95 % CI)**	**Highest P-Value** **(vs. Other Model/Timepoint)**
**Separate Analysis, 74 Gy Group**											
Week 1	Constant	−0.252	<0.001	0.801 (0.790––0.812)	0.872 (0.857––0.887)	0.698 (0.683––0.713)	<0.001^a^	0.766 (0.742––0.791)	0.770 (0.735––0.805)	0.851 (0.827––0.875)	0.152^b^
	W1: FOS1	−3.255	<0.001								
	W1: FOS11	−2.370	<0.001								
	W1: GLSZM5	−1.189	<0.001								
	W1: GLDZM14	4.162	<0.001								
	W1: GLDZM3	−1.086	<0.001								
	W1: GLDZM5	1.077	0.002								
	W1: GLDZM7	3.300	<0.001								
	W1: NGTDM3	−0.738	0.005								
	W1: NGLDM17	0.118	0.531								
	W1: NGLDM3	−0.850	<0.001								
**Separate Analysis, 60 Gy Group**											
Week 1	Constant	−0.149	0.026	0.833 (0.823––0.844)	0.783 (0.759––0.807)	0.808 (0.779––0.836)	<0.001^a^	0.734 (0.710––0.758)	0.805 (0.764––0.846)	0.723 (0.679––0.767)	<0.001^a^
	W1: FOS13	0.220	0.607								
	W1: GLSZM3	−0.054	0.827								
	W1: GLSZM5	0.697	0.026								
	W1: GLSZM6	−0.295	0.344								
	W1: GLSZM16	−0.818	<0.001								
	W1: GLDZM3	−0.379	0.192								
	W1: GLDZM5	−0.733	0.008								
	W1: GLDZM10	−0.882	0.080								
	W1: GLDZM14	1.741	0.003								
	W1: NGLDM7	−0.147	0.424								
**Cumulative Analysis, 74 Gy**											
Week 3	Constant	−0.274	0.006	0.858 (0.848––0.867)	0.789 (0.765––0.814)	0.829 (0.804––0.853)	0.450^c^	0.767 (0.744––0.791)	0.769 (0.736––0.803)	0.792 (0.758––0.825)	0.069^d^
	CT: FOS4	−0.133	0.892								
	CT: FOS22	−0.038	0.976								
	CT: FOS23	0.171	0.845								
	CT: GLCM7	−0.276	0.845								
	CT: GLDZM4	0.337	0.866								
	CT: GLDZM7	−0.280	0.538								
	CT: NGLDM15	−0.288	0.876								
	CT: NGLDM16	−0.071	0.957								
	W1: FOS11	−0.228	0.738								
	W1: GLDZM7	0.290	0.790								
	W1: GLDZM14	0.270	0.784								
	W1: NGTDM3	0.183	0.813								
	W2: FOS11	0.174	0.729								
	W2: GLSZM3	0.289	0.639								
	W3: GLCM6	−0.036	0.955								
	W3: GLRLM7	0.086	0.896								
	W3: NGTDM5	0.350	0.664								
**Cumulative Analysis, 60 Gy Group**											
Week 1	Constant	−0.033	0.773	0.731 (0.714––0.747)	0.685 (0.658––0.713)	0.814 (0.793––0.835)	0.994^e^	0.643 (0.616––0.670)	0.905 (0.882––0.927)	0.594 (0.559––0.629)	0.618^f^
	CT: FOS4	0.319	0.825								
	CT: GLCM25	−0.011	0.995								
	CT: GLSZM8	0.037	0.968								
	W1: GLDZM14	0.044	0.978								

Note:

a. Compared to all other models/timepoints.

b. Compared to the model at week 7 using week-level MVCT radiomic features extracted from treatment fractions 31–37 for the 74 Gy group in the separate analysis.

c. Compared to the model at week 4 using planning CT radiomics and week-level MVCT radiomic features extracted from treatment fractions 1–20 for the 74 Gy group in the cumulative analysis.

d. Compared to the model at week 5 using planning CT radiomics and week-level MVCT radiomic features extracted from treatment fractions 1–25 for the 74 Gy group in the cumulative analysis.

e. Compared to the model at week 4 using planning CT radiomics and week-level MVCT radiomic features extracted from treatment fractions 1–20 for the 60 Gy group in the cumulative analysis.

f. Compared to the model at week 0 using just the planning CT radiomics for the 60 Gy group in the cumulative analysis.

AUC: area under the curve; SEN: sensitivity; SPE: specificity; CI: confidence interval; CT: planning CT radiomic features; W1: week-level MVCT radiomic features extracted from treatment fractions 1–5; W2: week-level MVCT radiomic features extracted from treatment fractions 6–10; W3: week-level MVCT radiomic features extracted from treatment fractions 11–15.

## 4 Discussion

The aim of this study was to determine whether radiomic features derived at different time points during radiotherapy can improve prediction of rectal bleeding and thereby inform optimal timing of adaptive radiotherapy for toxicity reduction. Our analysis shows that radiomic features from the first week of treatment alone achieved strong predictive performance, indicating their substantial standalone utility. In particular, early features were not only predictive in isolation as shown in the separate analysis, but also provided a strong foundation for cumulative models that aggregated data across weeks. For the 74 Gy group, predictive performance plateaued after week 3, suggesting that extending model input beyond this point did not yield meaningful improvements, thereby identifying a practical and biologically relevant window for potential intervention. In contrast, while week 4 produced the highest training AUC in the 60 Gy group, its lower test performance likely reflects overfitting, highlighting the need to prioritise early, generalisable signals when identifying optimal re-planning time points.

The univariate analysis across all time points did not reveal any radiomic features with consistently statistically significant p-values below 0.05. This underlines the complex and evolving relationship between radiomic features and the manifestation of toxicity during radiotherapy, suggesting that radiomic features are influenced by both temporal anatomical changes and the biological response to radiation over time. This indicates the potential for radiomics to capture underlying biological processes that are not static but evolve in response to therapy. The dynamic nature of radiomic data highlights the challenges in using a single model for predicting radiation-induced toxicities and emphasises the need for adaptive strategies that can respond to changes during treatment [29,30].

Building on the univariate findings, logistic regression confirmed that early radiomic features can effectively predict toxicity risk, offering a valuable window for intervention. As their predictive relevance changes over time, adapting treatment in response to evolving radiomic patterns could support more personalised and effective radiotherapy. This adaptive strategy can ensure that treatment modifications are made when radiomic indicators signal a rise in the risk of toxicity. In practice, this approach could be integrated into routine treatment planning and monitoring systems, providing a continuous feedback loop that could help clinicians decide when and how to adjust treatment plans. This would ideally be supported by a framework enabling rapid model updates as new data become available, ensuring that the predictive models remain accurate and relevant throughout the treatment course [31,32].

Radiomic features often exhibit significant changes during radiotherapy, providing essential insights that can guide timely treatment adjustments [33]. In head and neck cancer, Berger et al. demonstrated that features extracted from daily MVCTs within the first half of treatment were most predictive of toxicity [34]. In PCa, Delgadillo et al. reported that delta-radiomic features from daily CBCTs could predict genitourinary toxicities and quality-of-life metrics as early as week 1 [35]. In lung cancer, van Timmeren et al. found most radiomic changes emerged by week 3, highlighting a key window for response assessment [36]. These studies collectively suggest that radiomic changes early in the treatment course are biologically informative and potentially actionable. Delaying adaptations beyond the halfway mark of the treatment risks missing the vital period when adjustments based on these changes can be effective. Moreover, early intervention allows for the correction of dosimetric discrepancies that could lead to increased side effects, thus preventing the accumulation of suboptimal doses as the treatment continues. This is particularly important in hypofractionation, which involves fewer treatment fractions and, consequently, fewer opportunities to adjust the treatment plan. In this context, Wu et al. showed that radiomic changes during a 5-fraction MR-guided radiotherapy were predictive of pathological complete response in rectal cancer [37]. Similarly, Jin et al. found that features extracted after just one or two fractions of stereotactic body radiotherapy (SBRT) for liver tumours could accurately predict local control [38]. These findings support the feasibility of leveraging radiomic information from early treatment fractions to inform adaptive strategies, even in ultra-hypofractionated settings.

In all the best-performing models, several consistently selected features were derived from first-order statistics (FOS), grey-level size zone matrix (GLSZM), and grey-level distance zone matrix (GLDZM). These features reflect not only voxel intensity distributions but also spatial heterogeneity in tissue structure within the rectal wall. For example, GLSZM and GLDZM quantify the size, number, and spatial distribution of homogeneous grey-level zones, which may be sensitive to early changes in tissue heterogeneity [15]. These changes could reflect evolving mucosal integrity or subclinical inflammation, both implicated in radiation-induced rectal bleeding [39,40].

Although classified as “mild” in CTCAE v4.03, grade 1 rectal bleeding is far from insignificant for patients [25]. In our cohort, over 30 % experienced grade ≥ 1 events, underscoring their relevance as an endpoint. Even small-volume or intermittent bleeding can cause distress, particularly when it appears months after treatment, as it often heightens fears of recurrence and acts as a persistent reminder of the cancer diagnosis. Such symptoms disrupt quality of life, are socially stigmatised, and may lead to further investigations to exclude recurrent or other pathology, creating additional burden for both patients and healthcare systems. In an era where advances in radiotherapy have reduced the incidence of severe toxicity, it is increasingly important to minimise the full spectrum of side effects, including low-grade events, to achieve the goal of truly personalised treatment.

One limitation of this study is the reduced sample size within each treatment group due to the inclusion of two different fractionation schedules. However, this design also represents a strength, as it enabled the evaluation of radiomic changes in both standard and moderately hypofractionated regimens, enhancing the clinical applicability of our findings. Nonetheless, the smaller sample sizes per group (82 and 58 for the training sets) may limit statistical power, particularly given the small effect sizes typically observed in radiomic features [41]. Although these features can capture subtle changes in tissue texture, shape, and intensity that may reflect early treatment response or emerging toxicity, such changes tend to be minor. As a result, larger datasets may be necessary to detect and validate these signals with greater confidence. Additionally, this study mainly focuses on specific time points, which may not fully capture the continuous dynamic changes in radiomic features throughout the treatment. Future studies could benefit from a more granular analysis that considers more frequent assessments of radiomic features [35,41]. Another notable limitation is the exclusion of daily accumulated dose information due to a lack of registration details between imaging sessions. Future research could improve upon this by integrating daily dose accumulation data, which would allow for a more comprehensive analysis of the relationship between radiomic changes and actual radiation exposure received by the tissue [23]. Another limitation is that shape- and size-based features, such as rectal wall volume, were not included. Although the rectal wall ROI was consistently defined, minor residual variations in size and shape might still influence dose distributions and thus affect toxicity outcomes. Future research should assess the added value of such features.

The ability of radiomics to capture temporal changes supports its use in creating more responsive and patient-specific radiotherapy plans. By continuously analysing radiomic data throughout treatment, clinicians can better predict when a patient is approaching a threshold of potential toxicity and adjust treatment parameters accordingly. Our findings suggest that radiomics could support a toxicity-driven approach to adaptive re-planning; in other words, even when the anatomical changes do not yet meet the clinical threshold to re-plan, radiomic features may reveal early signs of tissue sensitivity or evolving geometry that signal higher toxicity risks. This could complement the current ART practice, which primarily focuses on doses and geometry, by adding an additional layer focusing on normal tissue response. By leveraging the predictive power of radiomic features in this targeted and dynamic manner, the field of oncology can advance towards more personalised and precise cancer care.

## Declaration of competing interest

The authors declare that they have no known competing financial interests or personal relationships that could have appeared to influence the work reported in this paper.
