# Erratum to “Identifying the optimal time point for adaptive re-planning in prostate cancer radiotherapy to minimise rectal toxicity using normal tissue imaging biomarkers” [Phys. Imaging Radiat. Oncol. 36 (2025) 100850]

**DOI:** 10.1016/j.phro.2026.101061

**Published:** 2026-08-26

**Authors:** Zhuolin Yang, David J. Noble, Sarah Elliot, Leila Shelley, Thomas Berger, Raj Jena, Duncan B. McLaren, Neil G. Burnet, William H. Nailon

**Affiliations:** a Department of Oncology Physics, Edinburgh Cancer Centre, Western General Hospital, Crewe Road South, Edinburgh EH4 2XU, UK; b Institute for Imaging, Data and Communications, School of Engineering, University of Edinburgh, the King’s Buildings, Mayfield Road, Edinburgh EH9 3JL, UK; c Edinburgh Cancer Research, CRUK Scotland Centre, Institute of Genetics and Cancer, University of Edinburgh, Edinburgh, UK; d Department of Clinical Oncology, Edinburgh Cancer Centre, Western General Hospital, Crewe Road South, Edinburgh EH4 2XU, UK; e Centre for Medical Informatics, Usher Institute, University of Edinburgh, UK; f Radiation Oncology Department, Center Hospitalier Lyon Sud, Pierre Benite, France; g The University of Cambridge, Department of Oncology, Cambridge Biomedical Campus, Hills Road, Cambridge CB2 0QQ, UK; h Proton Centre, The Christie NHS Foundation Trust, Manchester, M20 4BX, UK; i School of Science and Engineering, the University of Dundee, Dundee DD1 4HN, UK

The **DeclarXXXation of CompXXXeting Interest Statement** for this paper is incomplete and should also have included the statement below:

*Given their role as Associate Editor, Professor Bill Nailon had no involvement in the peer-review of this article and has no access to information regarding its peer-review. Full responsibility for the editorial process for this article was delegated to another journal editor.*

The Publisher and authors apologise for this error.

