# Supplementary material for: Identifying the optimal time point for adaptive re-planning in prostate cancer radiotherapy to minimise rectal toxicity using normal tissue imaging biomarkers
Source: Phys Imaging Radiat Oncol. 2025 Oct 8;36:100850. doi: 10.1016/j.phro.2025.100850 (PMC12547930; doi:10.1016/j.phro.2025.100850)
Supplement: Supplementary Data 1 [file mmc1.pdf]

Supplementary Table S1

Parameter	Value	Number of Patients
Manufacture	Tomotherapy Incorporated	187
Manufacture's Model	Hi-Art	187
Slice Thickness	3 mm	187
Pixel Spacing	1.9531 mm	119
	2.1480 mm	52
	2.7340 mm	16
Rows/Columns	272	119
	256	68
Reconstruction Tool	Tomotherapy software version Tomo4_2_0_Apps 4.2.0.112	187

Supplementary Table S2

Parameter	Value	Number of Patients
Manufacture	Tomotherapy Incorporated	187
Manufacture's Model	Hi-Art	187
Slice Thickness<br>Pixel Spacing	6 mm for all fractions	178
	6 mm for 36 fractions &<br>4 mm for 1 fraction	4
	6 mm for 19 fractions &<br>4 mm for 1 fraction	2
	6 mm for 18 fractions &<br>4 mm for 2 fractions	3
Pixel Spacing	0.76 mm	187
Rows/Columns	512	187
Reconstruction Tool	Tomotherapy software version<br>Tomo4_2_0_Apps 4.2.0.112	187

Supplementary Table S3

**First order statistics (FOS)**	**Grey-level co-occurrence based features (GLCM)**
1. Mean	1. Joint maximum
2. Variance	2. Joint average
3. Skewness	3. Joint variance
4. Kurtosis	4. Joint entropy
5. Median	5. Difference average
6. Minimum	6. Difference variance
7. 10th percentile	7. Difference entropy
8. 90th percentile	8. Sum average
9. Maximum	9. Sum variance
10. Mode	10. Sum entropy
11. Interquartile range	11. Angular second moment
12. Range	12. Contrast
13. Mean absolute deviation	13. Dissimilarity
14. Robust mean absolute deviation	14. Inverse difference
15. Median absolute deviation	15. Normalised inverse difference
16. Coefficient of variation	16. Inverse difference moment
17. Quartile coefficient of dispersion	17. Normalised inverse difference moment
18. Entropy	18. Inverse variance
19. Uniformity	19. Correlation
20. Maximum histogram gradient	20. Autocorrelation
21. Maximum histogram gradient intensity	21. Cluster tendency
22. Minimum histogram gradient	22. Cluster shade
23. Minimum histogram gradient intensity	23. Cluster prominence
	24. Information correlation 1
	25. Information correlation 2
**Grey-level run-length based features (GLRLM)**	
1. Short runs emphasis	
2. Long runs emphasis	**Grey-level size zone based features (GLSZM)**
3. Low grey level run emphasis	1. Small zone emphasis

4. High grey level run emphasis	2. Large zone emphasis
5. Short run low grey level emphasis	3. Low grey level zone emphasis
6. Short run high grey level emphasis	4. High grey level zone emphasis
7. Long run low grey level emphasis	5. Small zone low grey level emphasis
8. Long run high grey level emphasis	6. Small zone high grey level emphasis
9. Grey level non-uniformity	7. Large zone low grey level emphasis
10. Normalised grey level non-uniformity	8. Large zone high grey level emphasis
11. Run length non-uniformity	9. Grey level non-uniformity
12. Normalised run length non-uniformity	10. Normalised grey level non-uniformity
13. Run percentage	11. Zone size non-uniformity
14. Grey level variance	12. Normalised zone size non-uniformity
15. Run length variance	13. Zone percentage
16. Run entropy	14. Grey level variance
	15. Zone size variance
	16. Zone size entropy
**Grey-level distance zone based features (GLDZM)**	
1. Small distance emphasis	
2. Large distance emphasis	**Neighbourhood grey-tone difference based features (NGTDM)**
3. Low grey level zone emphasis	1. Coarseness
4. High grey level zone emphasis	2. Contrast
5. Small distance low grey level emphasis	3. Busyness
6. Small distance high grey level emphasis	4. Complexity
7. Large distance low grey level emphasis	5. Strength
8. Large distance high grey level emphasis	
9. Grey level non-uniformity	
10. Normalised grey level non-uniformity	**Neighbouring grey-level dependence based features (NGLDM)**
11. Zone distance non-uniformity	1. Low dependence emphasis
12. Normalised zone distance non-uniformity	2. High dependence emphasis
13. Zone percentage	3. Low grey level count emphasis

14. Grey level variance	4. High grey level count emphasis
15. Zone distance variance	5. Low dependence low grey level emphasis
16. Zone distance entropy	6. Low dependence high grey level emphasis
	7. High dependence low grey level emphasis
	8. High dependence high grey level emphasis
	9. Grey level non-uniformity
	10. Normalised grey level non-uniformity
	11. Dependence count non-uniformity
	12. Normalised dependence count non-uniformity
	13. Dependence count percentage
	14. Grey level variance
	15. Dependence count variance
	16. Dependence count entropy
	17. Dependence count energy

Supplementary Table S4

74 Gy Group				60 Gy Group			
Timepoint	Feature Index	Feature Name	P-Value	Timepoint	Feature Index	Feature Name	P-Value
CT	GLDZM7	large distance low grey level emphasis	0.012	CT	FOS4	kurtosis	0.012
	NGLDM3	low grey level count emphasis	0.015		GLRLM9	grey level non-uniformity	0.032
	GLCM18	inverse variance	0.016		GLRLM11	run length non-uniformity	0.032
	NGLDM7	high dependence low grey level emphasis	0.016		GLRLM10	normalised grey level non-uniformity	0.040
	NGTDM5	strength	0.032		GLRLM12	normalised run length non-uniformity	0.040
	GLRLM7	long run low grey level emphasis	0.033		GLCM10	sum entropy	0.043
	GLCM3	joint variance	0.039		GLDZM12	normalised zone distance non-uniformity	0.052
	FOS16	coefficient of variation	0.040		GLDZM10	normalised grey level non-uniformity	0.055
	GLRLM3	low grey level run emphasis	0.043		GLSZM8	large zone high grey level emphasis	0.055
	FOS23	minimum histogram gradient intensity	0.049		GLSZM10	normalised grey level non-uniformity	0.058
MVCT Week 1	GLDZM14	grey level variance	0.120	MVCT Week 1	GLDZM14	grey level variance	0.138
	FOS11	interquartile range	0.126		GLRLM14	grey level variance	0.166
	GLSZM16	zone size entropy	0.129		FOS13	mean absolute deviation	0.181
	GLCM10	sum entropy	0.129		FOS14	robust mean absolute deviation	0.186
	NGTDM3	busyness	0.133		GLSZM14	grey level variance	0.186
	GLRLM9	grey level non-uniformity	0.135		GLCM3	joint variance	0.186
	GLRLM11	run length non-uniformity	0.135		FOS2	variance	0.214
	GLSZM14	high grey level zone emphasis	0.142		NGLDM14	grey level variance	0.214
	FOS20	maximum histogram gradient	0.169		FOS15	median absolute deviation	0.262
	NGLDM9	grey level non-uniformity	0.189		GLSZM5	small zone low grey level emphasis	0.272
MVCT Week 2	GLSZM3	low grey level zone emphasis	0.008	MVCT Week 2	FOS18	entropy	0.077
	GLRLM3	low grey level run emphasis	0.026		FOS22	minimum histogram gradient	0.082
	GLRLM5	short run low grey level emphasis	0.047		NGLDM9	grey level non-uniformity	0.088
	GLDZM3	low grey level zone emphasis	0.048		GLRLM10	normalised grey level non-uniformity	0.091
	NGTDM5	strength	0.053		GLRLM12	normalised zone distance non-uniformity	0.091

	NGLDM3	low grey level count emphasis	0.062		FOS19	uniformity	0.092
	FOS16	coefficient of variation	0.123		NGLDM10	normalised grey level non-uniformity	0.092
	FOS17	quartile coefficient of dispersion	0.208		FOS17	quartile coefficient of dispersion	0.099
	GLDZM8	large distance high grey level emphasis	0.208		FOS4	kurtosis	0.104
	GLRLM7	long run low grey level emphasis	0.229		FOS11	interquartile range	0.109
MVCT Week 3	NGTDM5	strength	0.042	MVCT Week 3	GLRLM3	low grey level run emphasis	0.057
	GLRLM3	low grey level run emphasis	0.059		NGLDM3	low grey level count emphasis	0.107
	GLDZM8	large distance high grey level emphasis	0.080		FOS8	90th percentile	0.143
	NGLDM3	low grey level count emphasis	0.122		GLDZM14	grey level variance	0.145
	FOS16	coefficient of variation	0.138		GLRLM14	grey level variance	0.156
	GLRLM7	long run low grey level emphasis	0.148		GLDZM3	low grey level zone emphasis	0.165
	GLCM10	sum entropy	0.169		GLRLM5	short run low grey level emphasis	0.166
	GLCM3	joint variance	0.190		FOS10	mode	0.176
	FOS17	quartile coefficient of dispersion	0.216		NGTDM1	coarseness	0.180
	NGLDM7	high dependence low grey level emphasis	0.219		NGTDM2	contrast	0.189
MVCT Week 4	GLSZM3	low grey level zone emphasis	0.021	MVCT Week 4	FOS11	interquartile range	0.020
	NGTDM5	strength	0.032		FOS14	robust mean absolute deviation	0.025
	GLRLM7	long run low grey level emphasis	0.034		FOS15	median absolute deviation	0.036
	GLCM3	joint variance	0.034		GLRLM9	grey level non-uniformity	0.062
	GLRLM14	grey level variance	0.049		GLRLM11	run length non-uniformity	0.062
	GLRLM3	low grey level run emphasis	0.055		GLSZM16	zone size entropy	0.071
	GLDZM14	grey level variance	0.056		GLCM10	sum entropy	0.088
	FOS13	mean absolute deviation	0.062		NGLDM17	dependence count energy	0.099
	FOS2	variance	0.064		NGTDM3	busyness	0.132
	NGLDM14	grey level variance	0.064		FOS13	mean absolute deviation	0.168
MVCT Week 5	GLDZM8	large distance high grey level emphasis	0.239				
	GLSZM6	small zone high grey level emphasis	0.252				
	NGTDM5	strength	0.285				
	NGLDM7	high dependence low grey level emphasis	0.303				
	NGLDM17	dependence count energy	0.311				

	GLRLM7	long run low grey level emphasis	0.346
	GLCM10	sum entropy	0.354
	GLSZM7	large zone low grey level emphasis	0.373
	NGLDM6	low dependence high grey level emphasis	0.384
	GLCM6	difference variance	0.406
MVCT Week 6	GLRLM3	low grey level run emphasis	0.031
	NGLDM3	low grey level count emphasis	0.061
	GLRLM7	long run low grey level emphasis	0.114
	GLRLM5	short run low grey level emphasis	0.117
	GLDZM3	low grey level zone emphasis	0.155
	NGTDM5	strength	0.161
	NGLDM7	high dependence low grey level emphasis	0.163
	GLDZM14	grey level variance	0.174
	GLSZM3	low grey level zone emphasis	0.214
	GLSZM7	large zone low grey level emphasis	0.214
MVCT Week 7	NGTDM5	strength	0.075
	FOS16	coefficient of variation	0.079
	GLRLM3	low grey level run emphasis	0.099
	FOS17	quartile coefficient of dispersion	0.123
	NGLDM3	low grey level count emphasis	0.125
	GLRLM5	short run low grey level emphasis	0.137
	FOS7	10th percentile	0.164
	GLCM3	joint variance	0.174
	GLSZM10	normalised grey level non-uniformity	0.185
	FOS14	robust mean absolute deviation	0.196

Supplementary Table S5

74 Gy Group				60 Gy Group			
**Timepoint**	**Feature Index**	**Feature Name**	**P-Value**	**Timepoint**	**Feature Index**	**Feature Name**	**P-Value**
CT	GLDZM7	large distance low grey level emphasis	0.012	CT	FOS4	kurtosis	0.012
	NGLDM3	low grey level count emphasis	0.015		GLCM10	sum entropy	0.043
	GLCM18	inverse variance	0.016		GLDZM12	normalised zone distance non-uniformity	0.052
	FOS23	minimum histogram gradient intensity	0.049		GLSZM8	large zone high grey level emphasis	0.055
	GLCM6	difference variance	0.053		GLCM25	information correlation 2	0.060
	FOS15	median absolute deviation	0.070		FOS3	skewness	0.071
	GLDZM4	high grey level zone emphasis	0.079		FOS15	median absolute deviation	0.116
	GLRLM16	run entropy	0.111		GLSZM5	small zone low grey level emphasis	0.171
	GLCM17	normalised inverse difference moment	0.148		GLSZM12	normalised zone size non-uniformity	0.214
	GLDZM9	grey level non-uniformity	0.172		GLCM22	cluster shade	0.233
	GLDZM2	large distance emphasis	0.187		GLDZM6	small distance high grey level emphasis	0.233
	FOS10	mode	0.206		FOS22	minimum histogram gradient	0.265
	FOS4	kurtosis	0.249		NGLDM3	low grey level count emphasis	0.328
	GLCM7	difference entropy	0.278		GLCM7	difference entropy	0.353
	NGLDM15	dependence count variance	0.325		GLCM12	contrast	0.357
	GLSZM12	normalised zone size non-uniformity	0.348		GLCM6	difference variance	0.374
	GLDZM5	small distance low grey level emphasis	0.491		FOS8	90th percentile	0.437
	NGLDM5	low dependence low grey level emphasis	0.531		FOS21	maximum histogram gradient intensity	0.591
	FOS8	90th percentile	0.636		GLDZM7	large distance low grey level emphasis	0.648
	FOS22	minimum histogram gradient	0.661		GLDZM5	small distance low grey level emphasis	0.659
	GLSZM5	small zone low grey level emphasis	0.687		NGLDM5	low dependence low grey level emphasis	0.739
	NGLDM16	dependence count entropy	0.695		GLSZM9	grey level non-uniformity	0.743
	GLCM22	cluster shade	0.710		GLCM18	inverse variance	0.754
MVCT Week 1	GLDZM14	grey level variance	0.120		FOS23	minimum histogram gradient intensity	0.816
	FOS11	interquartile range	0.126		GLDZM8	large distance high grey level emphasis	0.943

	NGTDM3	busyness	0.133
	GLDZM7	large distance low grey level emphasis	0.308
	FOS1	mean	0.460
	GLDZM3	low grey level zone emphasis	0.519
	NGLDM17	dependence count energy	0.629
	NGLDM3	low grey level count emphasis	0.713
	GLSZM5	small zone low grey level emphasis	0.740
	GLDZM5	small distance low grey level emphasis	0.947
MVCT Week 2	GLSZM3	low grey level zone emphasis	0.008
	GLRLM3	low grey level run emphasis	0.026
	FOS16	coefficient of variation	0.123
	GLRLM7	long run low grey level emphasis	0.229
	FOS11	interquartile range	0.278
	GLDZM14	grey level variance	0.320
	GLSZM6	small zone high grey level emphasis	0.492
	GLDZM5	small distance low grey level emphasis	0.553
	FOS20	maximum histogram gradient	0.607
	GLDZM7	large distance low grey level emphasis	0.646
	GLSZM5	small zone low grey level emphasis	0.676
	GLSZM16	zone size entropy	0.842
MVCT Week 3	NGTDM5	strength	0.042
	GLDZM8	large distance high grey level emphasis	0.080
	GLRLM7	long run low grey level emphasis	0.148
	FOS17	quartile coefficient of dispersion	0.216
	GLRLM5	short run low grey level emphasis	0.260
	GLSZM6	small zone high grey level emphasis	0.269
	GLCM6	difference variance	0.336
	GLSZM3	low grey level zone emphasis	0.346
	GLDZM7	large distance low grey level emphasis	0.403
	GLSZM14	grey level variance	0.621

MVCT Week 1	GLDZM14	grey level variance	0.138
	FOS13	mean absolute deviation	0.181
	GLSZM5	small zone low grey level emphasis	0.272
	GLDZM10	normalised grey level non-uniformity	0.308
	GLSZM3	low grey level zone emphasis	0.368
	GLDZM5	small distance low grey level emphasis	0.395
	GLSZM6	small zone high grey level emphasis	0.466
	GLSZM16	zone size entropy	0.506
	NGLDM7	high dependence low grey level emphasis	0.516
	GLDZM3	low grey level zone emphasis	0.585
	GLDZM16	zone distance entropy	0.586
	GLDZM8	large distance high grey level emphasis	0.816
MVCT Week 2	FOS18	entropy	0.077
	FOS11	interquartile range	0.109
	GLRLM5	short run low grey level emphasis	0.119
	GLSZM14	grey level variance	0.134
	GLSZM7	large zone low grey level emphasis	0.147
	GLSZM6	small zone high grey level emphasis	0.152
	GLSZM16	zone size entropy	0.232
	GLDZM8	large distance high grey level emphasis	0.245
	GLSZM3	low grey level zone emphasis	0.396
	GLDZM5	small distance low grey level emphasis	0.431
	GLCM3	joint variance	0.671
MVCT Week 3	GLRLM3	low grey level run emphasis	0.057
	FOS8	90th percentile	0.143
	NGTDM1	coarseness	0.180
	GLCM3	Joint variance	0.220
	NGLDM16	dependence count entropy	0.344
	GLSZM3	low grey level zone emphasis	0.393
	FOS18	entropy	0.564

	FOS22	minimum histogram gradient	0.815
MVCT Week 4	GLSZM3	low grey level zone emphasis	0.021
	NGTDM5	strength	0.032
	GLCM3	joint variance	0.034
	GLDZM14	grey level variance	0.056
	GLDZM5	small distance low grey level emphasis	0.168
	FOS22	minimum histogram gradient	0.315
	GLDZM8	large distance high grey level emphasis	0.330
	GLSZM10	normalised grey level non-uniformity	0.359
	GLCM10	sum entropy	0.548
	GLSZM5	small zone low grey level emphasis	0.725
	GLDZM7	large distance low grey level emphasis	0.783
MVCT Week 5	3	large distance high grey level emphasis	0.239
	GLSZM6	small zone high grey level emphasis	0.252
	NGTDM5	strength	0.285
	NGLDM7	high dependence low grey level emphasis	0.303
	GLCM10	sum entropy	0.354
	GLCM14	inverse difference	0.424
	GLDZM5	small distance low grey level emphasis	0.628
	GLDZM3	low grey level zone emphasis	0.705
	NGTDM1	coarseness	0.809
	NGTDM3	busyness	0.927
MVCT Week 6	GLRLM3	Low grey level run emphasis	0.031
	GLRLM7	long run low grey level emphasis	0.114
	GLDZM3	low grey level zone emphasis	0.155
	GLSZM14	grey level variance	0.239
	FOS20	maximum histogram gradient	0.262
	FOS23	minimum histogram gradient intensity	0.308
	FOS15	median absolute deviation	0.318
	GLSZM5	small zone low grey level emphasis	0.491

	GLSZM16	zone size entropy	0.718
	GLSZM6	small zone high grey level emphasis	0.785
	GLDZM5	small distance low grey level emphasis	0.917
	FOS11	interquartile range	1.000
MVCT Week 4	FOS11	interquartile range	0.020
	GLSZM16	zone size entropy	0.071
	NGTDM3	busyness	0.132
	GLCM3	Joint variance	0.208
	GLSZM3	low grey level zone emphasis	0.278
	GLSZM5	small zone low grey level emphasis	0.297
	FOS17	quartile coefficient of dispersion	0.357
	NGTDM5	strength	0.471
	FOS23	minimum histogram gradient intensity	0.476
	GLRLM5	short run low grey level emphasis	0.552
	GLDZM14	grey level variance	0.779
	GLSZM6	small zone high grey level emphasis	0.930
	FOS20	maximum histogram gradient	0.936

	GLSZM10	normalised grey level non-uniformity	0.520
	GLDZM7	large distance low grey level emphasis	0.551
MVCT Week 7	NGTDM5	strength	0.075
	FOS17	quartile coefficient of dispersion	0.123
	GLRLM5	short run low grey level emphasis	0.137
	GLCM3	joint variance	0.174
	GLDZM8	large distance high grey level emphasis	0.243
	GLSZM3	low grey level zone emphasis	0.334
	GLSZM7	large zone low grey level emphasis	0.451
	FOS4	kurtosis	0.665
	GLDZM5	small distance low grey level emphasis	0.878
